# Dynamic T‐Cell Reprogramming Modulates the Treatment Outcome of Neoadjuvant Immunochemotherapy in Non‐Small‐Cell Lung Cancer

**DOI:** 10.1002/mco2.70690

**Published:** 2026-03-18

**Authors:** Rui Jin, Anhao Tian, Weina Lu, Qiyuan Wang, Xiuzhen Li, Sai Zhang, Guanxin Xu, Kai Zhu, Peng Li, Jianan Li, Wei Chen, Weiwei Yin, Wen Li, Yang Xia

**Affiliations:** ^1^ Key Laboratory of Respiratory Disease of Zhejiang Province Department of Respiratory and Critical Care Medicine Second Affiliated Hospital of Zhejiang University School of Medicine Hangzhou Zhejiang China; ^2^ Department of Neurosurgery Second Affiliated Hospital of Zhejiang University School of Medicine Hangzhou Zhejiang China; ^3^ Cancer Centre Zhejiang University Hangzhou Zhejiang China; ^4^ Department of Radiology Second Affiliated Hospital of Zhejiang University School of Medicine Hangzhou Zhejiang China; ^5^ Department of Pathology Second Affiliated Hospital of Zhejiang University School of Medicine Hangzhou Zhejiang China; ^6^ Department of Thoracic Surgery Second Affiliated Hospital of Zhejiang University School of Medicine Hangzhou Zhejiang China; ^7^ Wenzhou Medical University Wenzhou Zhejiang China; ^8^ Zhejiang Puluoting Health Technology Co., Ltd Hangzhou Zhejiang China; ^9^ College of Biomedical Engineering and Instrument Science Key Laboratory for Biomedical Engineering of Ministry of Education Zhejiang University Hangzhou Zhejiang China; ^10^ School of Basic Medical Science Zhejiang University Hangzhou Zhejiang China; ^11^ Zhejiang Provincial Key Laboratory of Cardio‐Cerebral Vascular Detection Technology and Medicinal Effectiveness Appraisal College of Biomedical Engineering and Instrument of Science Zhejiang University Hangzhou Zhejiang China

**Keywords:** immunochemotherapy, neoadjuvant, non‐small‐cell lung cancer (NSCLC), T cell

## Abstract

Although immunochemotherapy sheds light on neoadjuvant strategies, about two‐thirds of patients still respond poorly to perioperative chemoimmunotherapy. Hence, it is crucial to investigate the underlying response mechanism to improve the prognosis of these patients. In this study, we utilized paired pre‐ and post‐neoadjuvant immunochemotherapy samples from non‐small‐cell lung cancer (NSCLC) patients with single‐cell RNA and T‐cell receptor (TCR) sequencing to characterize the dynamic changes of T cells in tumor microenvironment. Within nine enrolled patients with distinct pathological assessments, we identified bi‐directional mechanisms associated with their pathological responsiveness. One is mediated by a batch of CD8^+^ T‐cell subsets such as effector memory T cells (Tem), effector T cells (Teff), tissue‐resident memory T cells (Trm), and exhausted T cells (Tex), exhibiting higher TCR clonality and diversity in responders. CD8^+^ Tem cells with both novel and pre‐existing TCR clonal expansion patterns particularly contributed to improved pathological responses. The other mechanism is through inhibitory Tregs, which showed more novel clonal expansion and enhanced functional profiles in nonresponsive tumors. In conclusion, our findings proposed the bidirectional characteristics of T‐cell dynamics for in‐depth interpretation of responding mechanisms to neoadjuvant immunochemotherapy of NSCLC.

## Graphical abstract

We explored the responding mechanisms associated with pathological responsiveness to neoadjuvant immunochemotherapy. CD8+ T‐cell subsets, including effect memory T (Tem), effector T (Teff), resident memory T (Trm), as well as exhausted T cells (Tex), showed higher clonality and diversity in responders, and both novel and pre‐existing clonal expansion of CD8+ Tem cells contributed to improved pathological response. By contrast, the inhibitory Treg cells presented enhanced activities with higher novel clones in nonresponders, and more specifically, novel clonal generation of CD4+ Treg cells diminished the therapeutic efficacy.

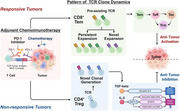

## 1 Introduction

Approximately one‐quarter of patients with non‐small‐cell lung cancer (NSCLC) present with resectable disease, typically ranging from stage I to stage IIIA and selected cases of stage IIIB. However, surgery alone often fails to achieve a cure, particularly for patients with stage II–III disease, where postoperative recurrence rates can reach as high as 40%–60% [1]. This limitation underscores the pressing need for integrated perioperative therapy strategies. The dual objectives of perioperative therapy are to improve resectability by downstaging tumors through neoadjuvant approaches and to reduce recurrence by eliminating micrometastases with adjuvant treatments, ultimately aiming to enhance cure rates.

Traditional methods, such as perioperative chemotherapy, may partially improve the chances of achieving complete resection. However, their impact on 5‐year survival rates is modest, yielding only a 5% absolute improvement [2]. This limited benefit highlights the crucial necessity for more effective treatment options.

Immunotherapy has introduced new possibilities for neoadjuvant strategies. Notably, the CheckMate 159 trial marked a significant advancement, demonstrating a major pathological response (MPR) rate of 45% [3] and a 24‐month recurrence‐free survival (RFS) rate of 69% [4]. Subsequent trials have explored the efficacy of neoadjuvant immunochemotherapy, with robust evidence from the pivotal CheckMate 816 trial and several studies on perioperative immune checkpoint inhibition [5, 6, 7]. These investigations have established the superiority of neoadjuvant immunochemotherapy in terms of pathological efficacy and long‐term survival benefits compared to neoadjuvant chemotherapy alone. Despite these accumulating clinical evidences supporting the significant role of neoadjuvant immunotherapy, approximately two‐thirds of patients who do not achieve a major pathological response (non‐MPR) outcome still do not benefit from this approach [5]. This stark reality underscores the urgent need to elucidate the underlying mechanisms of treatment response.

While preliminary studies have begun to address the mechanisms of neoadjuvant immunotherapy in the context of NSCLC, several key questions remain unanswered [8, 9]. Caushi and colleagues identified the critical role of mutation‐associated neoantigen (MANA) specific tissue‐resident memory T cells in regulating the responsiveness to neoadjuvant immune monotherapy [10]. However, it remains unclear whether these T cells play a comparable role in the context of neoadjuvant immunochemotherapy. Recent studies have utilized paired blood samples or unpaired tissue specimens to interpret the microenvironmental dynamics during neoadjuvant immunochemotherapy [11, 12]. Although neoadjuvant immunotherapy has been shown to trigger both local and systemic immune responses in solid tumors, significant heterogeneity exists between intratumor infiltrating immune cells and circulating immune cells [13, 14]. Furthermore, when analyzing tumor tissue samples from different patients at varying treatment timepoints, it becomes challenging to distinguish between pre‐existing patient‐induced heterogeneity and therapy‐induced heterogeneity. This complexity makes it nearly impossible to disentangle genuine treatment effects from the substantial baseline heterogeneity among individuals.

To address these concerns, we utilized a unique set of paired pre‐ and post‐treatment biopsies from NSCLC patients undergoing neoadjuvant immunochemotherapy. By applying paired single‐cell RNA sequencing (scRNA‐seq) and T‐cell receptor (TCR) sequencing to these samples, we conducted a precise analysis to characterize the dynamic changes of T cells during immunochemotherapy and their association with pathological responsiveness. Our study elucidates the mechanisms of T‐cell immune reprogramming during neoadjuvant immunochemotherapy, aiming to provide novel insights into overcoming immune checkpoint blockade resistance and laying a rational foundation for future therapeutic optimization.

## 2 Results

### 2.1 T‐Cell Dynamics in NSCLC Tumors Responsive to Neoadjuvant Immunochemotherapy

**FIGURE 1:**
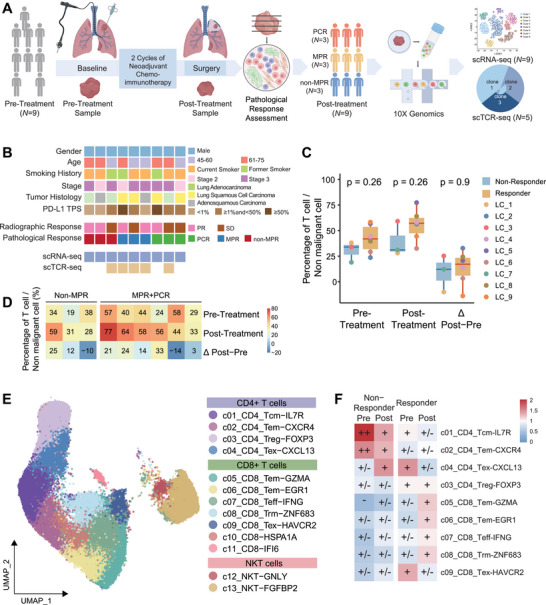
T‐cell dynamics in NSCLC tumors responsive to neoadjuvant immunochemotherapy. (A) Scheme diagram of the overall study design. Single‐cell RNA sequencing was applied to whole cells derived from nine paired lung cancer samples from NSCLC patients pre‐ and post‐neoadjuvant immunochemotherapy. T‐cell receptor (TCR) sequencing was applied to T cells from 10 paired samples. (B) Clinical information and pathological response of nine patients. (C) Box plot of the percentage of T cells in nonmalignant cells in nonresponsive tumors (blue) and responsive tumors (orange) at pre‐, post‐, and differences between post‐ and pre‐neoadjuvant immunochemotherapy. Center line indicates the median value, lower and upper hinges represent the 25th and 75th percentiles, respectively, and whiskers denote 1.5× interquartile range. Each dot corresponds to one patient. (D) Heatmap of percentage of T cells in all cells in each patient at pre‐, post‐, and differences between post‐ and pre‐ neoadjuvant immunochemotherapy. The color density indicates the percentage or percentage difference of T cells in all cells. (E) UMAP visualization of 50,563 single T cells from nine paired lung cancer samples, showing the formation of 14 T clusters, including four CD4^+^ T subgroups (c01_CD4_Tn‐CCR7, c02_CD4_Tem‐CXCR4, c03_CD4_Treg‐FOXP3, and c04_CD4_Tex‐CXCL13), seven CD8^+^ T subgroups (c05_CD8_Tem‐GZMA, c06_CD8_Tem‐EGR1, c07_CD8_Teff‐IFNG, c08_CD8_Trm‐ZNF683, c09_CD8_Tex‐HAVCR2, c10_CD8‐HSPA1A, and c11_CD8‐IFI6), two NKT subgroups (c12_NKT‐GNLY and c13_NKT‐FGFBP2). Each dot corresponds to a single cell, colored by cell cluster. (F) Tissue preference of T‐cell clusters at pre‐, post‐, and differences between post‐ and pre‐neoadjuvant immunochemotherapy, revealed by Ro/e (ratio of observed cell number to expected cell number).

To investigate how neoadjuvant immunochemotherapy reshapes the tumor microenvironment in NSCLC, we conducted a prospective, single‐arm study. Nine patients with treatment‐naïve, stage IIB–IIIB NSCLC were enrolled at the Second Affiliated Hospital of Zhejiang University School of Medicine between March 2021 and September 2021. All patients received two cycles of camrelizumab plus platinum‐based chemotherapy prior to surgery (Figure 1A). Based on postoperative pathological assessment, three patients achieved a pathological complete response (PCR) with no residual tumor in resected lymph nodes, three achieved a major pathologic response (MPR), and three were classified as non‐MPR (Figure 1B, Section 4). Detailed demographics and clinical characteristics are summarized in Table 1 and Figure 1B, with no significant baseline differences between responders and nonresponders. Nine paired lung tumor biopsy samples were collected before and after neoadjuvant immunochemotherapy and analyzed using scRNA‐seq (Figure 1A). Tumors from non‐MPR patients were defined as nonresponsive, whereas PCR and MPR tumors were considered responsive.

**TABLE 1:** Baseline demographic and disease characteristics of patients.

Characteristics	Overall (*N* = 9)	Responder (*N* = 6)	Nonresponder (*N* = 3)	*p* value
Age, year (range)	64 (45–74)	61.5 (56–74)	66 (45–69)	0.905^a^
Sex, no. (%)				
Male	9 (100.0)	6 (100.0)	3 (100.0)	
Smoking history, no. (%)				
Current and former smoker	9 (100.0)	6 (100.0)	3 (100.0)	
Tumor histology, no. (%)				0.222^b^
Adenocarcinoma	3 (33.3)	1 (16.7)	2 (66.7)	
Squamous cell carcinoma	4 (44.4)	4 (66.7)	0 (0.0)	
Adenosquamous carcinoma	2 (22.2)	1 (16.7)	1 (33.3)	
Tumor location, no. (%)				>0.999^b^
Central	4 (44.4)	3 (50.0)	1 (33.3)	
Peripheral	5 (55.6)	3 (50.0)	2 (66.7)	
Baseline disease stage, no. (%)				>0.999^b^
Stage II	3 (33.3)	2 (33.3)	1 (33.3)	
Stage III	6 (66.7)	4 (66.7)	2 (66.7)	
Baseline T stage, no. (%)				0.381^b^
T2	3 (33.3)	1 (16.7)	2 (66.7)	
T3	4 (44.4)	3 (50.0)	1 (33.3)	
T4	2 (22.2)	2 (33.3)	0 (0.0)	
Baseline N stage, no. (%)				0.190^b^
N0	1 (11.1)	1 (16.7)	0 (0.0)	
N1	5 (55.6)	4 (66.7)	1 (33.3)	
N2	3 (33.3)	1 (16.7)	2 (66.7)	
Baseline PD‐L1 TPS (tumor proportion score), no. (%)				0.242^b^
<1%	3 (33.3)	1 (16.7)	2 (66.7)	
≥ 1% and < 50%	4 (44.4)	3 (50.0)	1 (33.3)	
≥ 50%	2 (22.2)	2 (33.3)	0 (0.0)	

^a^ Mann–Whitney *U* test.

^b^ Fisher's exact test.

Following quality control, 140,711 high‐quality cells were retained for downstream analysis (Figure S1, Section 4). Seven major cell types were identified: epithelial cells, T cells, B cells, myeloid cells, endothelial cells, fibroblasts, and proliferating cells (Figure S2, Table S1). Epithelial cells were further classified into malignant and nonmalignant subsets using inferCNV [15]. These major cell populations displayed distinct distribution patterns depending on treatment efficacy and timepoint (Figure S3). Notably, responsive tumors showed consistently higher proportions of T cells than nonresponsive tumors at both pre‐ and post‐treatment timepoints and exhibited a greater elevation in T‐cell abundance following therapy (Figure 1C,D). These observations highlight the dynamic enrichment of T cells in response to neoadjuvant immunochemotherapy and motivated a focused analysis of T‐cell phenotypes and their association with therapeutic outcomes.

We identified 50,563 T cells and clustered them into 14 subpopulations using supervised cell‐type annotation (Figure 1E, Section 4). These included four CD4^+^ T‐cell subgroups (c01_CD4_Tn‐CCR7, c02_CD4_Tem‐CXCR4, c03_CD4_Treg‐FOXP3, and c04_CD4_Tex‐CXCL13), seven CD8^+^ T‐cell subgroups (c05_CD8_Tem‐GZMA, c06_CD8_Tem‐EGR1, c07_CD8_Teff‐IFNG, c08_CD8_Trm‐ZNF683, c09_CD8_Tex‐HAVCR2, c10_CD8‐HSPA1A, and c11_CD8‐IFI6), and two NKT subgroups (c12_NKT‐GNLY and c13_NKT‐FGFBP2) (Figure S4).

Distinct enrichment patterns of these T‐cell subpopulations were associated with treatment responses (Figure 1F). Specifically, post‐treatment responsive tumors were enriched for c05_CD8_Tem‐GZMA, c06_CD8_Tem‐EGR1, c07_CD8_Teff‐IFNG, and c08_CD8_Trm‐ZNF683 cells. In contrast, c03_CD4_Treg‐FOXP3 cells were significantly enriched in nonresponsive post‐treatment tumors (Figure 1F). Together, these findings suggest that divergent pathological responses to neoadjuvant immunochemotherapy may be driven by distinct immune mechanisms—one characterized by activated CD8^+^ T‐cell programs and the other by suppressive regulatory T cell (Treg)‐mediated pathways—warranting deeper mechanistic investigation.

### 2.2 Enriched Clonal Expansion of CD8^+^ T Cells and CD4^+^ Treg Cells Induced by Neoadjuvant Immunochemotherapy in Responsive and Nonresponsive Tumors

To further investigate T‐cell dynamics in response to neoadjuvant immunochemotherapy, we performed single‐cell TCR sequencing on paired tissue samples from five out of the nine NSCLC patients. After data preprocessing, we identified 19,042 T cells with productive TCR alpha/beta chains and 11,322 effective TCR clonotypes with matched alpha and beta chain pairs, enabling us to track T‐cell clonal compositions and their potential associations with therapeutic efficacy.

**FIGURE 2:**
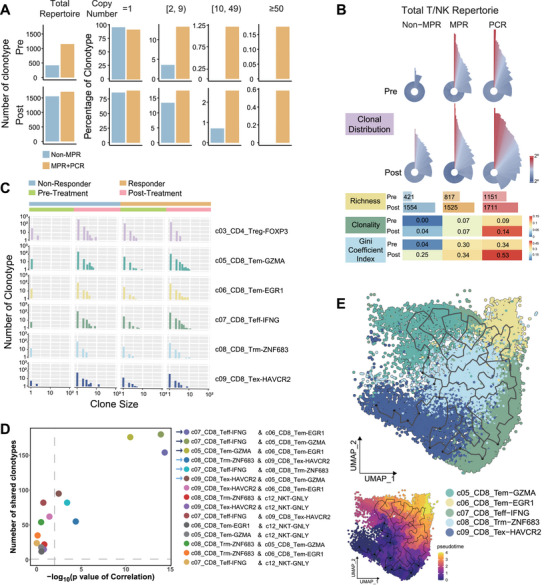
Enriched clonal expansion of CD8+ T cells and CD4+ Treg cells induced by neoadjuvant immunochemotherapy in responsive and nonresponsive tumors. (A) Bar plot of number of TCR clonotypes and proportion of clonotypes with specific copy numbers among all T cells in nonresponsive (blue) and responsive tumors (orange). (B) TCR measurements of clonal distribution of all T cells stratified by neoadjuvant efficacy and treatment timepoint. The rose plot (top panel) shows the clonal distribution of each subgroup. In each sector, the radius length and color density represent the clone size, and the angle represents the proportion of the clone. The bar plot (middle panel) shows the richness of clonotypes in each subgroup. The heatmap (bottom panel) represents the clonal diversity measures in each subgroup. The color density indicates clonality and Gini coefficient index. (C) Histogram of TCR clonal distribution of five CD8^+^ T subgroups and CD4^+^ Tregs stratified by neoadjuvant efficacy and treatment time point. In each bar, the horizontal coordinate represents the clone size (copy number of the clonotype) of these clonotypes and the vertical coordinate represents the number of these clonotypes. (D) Correlation analysis of TCRs shared across CD8^+^ T subgroups. Y‐axis indicates the number of shared clonotypes, and X‐axis indicates *p* value of correlation. (E) *Monocle 3* analysis of trajectory of CD8^+^ T‐cell state transition. UMAP is colored by T‐cell subgroups.

To assess the quantity and diversity of TCR repertoires in different response groups, we compared repertoire richness and clonal diversity at both pre‐ and post‐treatment stages. We observed an elevated number of total TCR clonotypes following neoadjuvant therapy (Figure 2A). Notably, in the non‐MPR patient, the total number of TCR clonotypes increased by approximately threefold after treatment, though most of the expansion occurred in nonexpanded clones (copy number = 1) (Figure 2A). In contrast, for responders, although the increase in TCR clonotypes was around 50%, the expansion was largely in expanded clones (Figure 2A). In particular, highly expanded clonotypes (copy number > 10 or > 50) increased by two‐ to fourfold in responsive patients at post‐treatment, a phenomenon not observed in the non‐MPR patient (Figure 2A).

We then visualized TCR clonal distributions using Roseplot, which revealed more pronounced TCR clonality and expansion in post‐treatment responsive tumors compared to nonresponsive tumors (Figure 2B, upper panel). This result was further confirmed by calculating TCR clonality and the Gini coefficient index [16] for different sample groups. Post‐treatment PCR samples exhibited the highest clonality and Gini index values, while non‐MPR samples had the lowest levels of T‐cell expansion (Figure 2C), suggesting that the extent of T‐cell expansion is associated with the efficacy of neoadjuvant therapy. Consequently, we examined the expansion of different T‐cell subsets and observed preferential expansion of several CD8^+^ T‐cell subsets (e.g., c05_CD8_Tem‐GZMA, c06_CD8_Tem‐EGR1, c07_CD8_Teff‐IFNG, c08_CD8_Trm‐ZNF683, and c09_CD8_Tex‐HAVCR2) in responsive tumors, while CD4^+^ Treg cells were specifically expanded in nonresponsive tumors (Figure 2C, Figure S5A).

To explore the relationship between these expanded CD8^+^ T‐cell subsets, we examined shared TCR clonotypes (Section 4) between subsets. In post‐treatment PCR tumor cells, correlation analysis revealed that three CD8^+^ clusters—two effector memory (c05 and c06) and one effector (c07) subsets—showed significant clonal sharing (Figure 2D). Additionally, we identified significant clonal sharing between these subsets and the resident memory (c08) and exhaustion (c09) subsets (Figure 2D). Trajectory analysis (Section 4) suggested a potential progression from two effector memory T‐cell subsets to effector T cells, followed by transition to resident memory T cells and ultimately to exhausted T cells (Figure 2E). This pattern was further supported by RNA velocity and Slingshot (Figure S5B,C).

**FIGURE 3:**
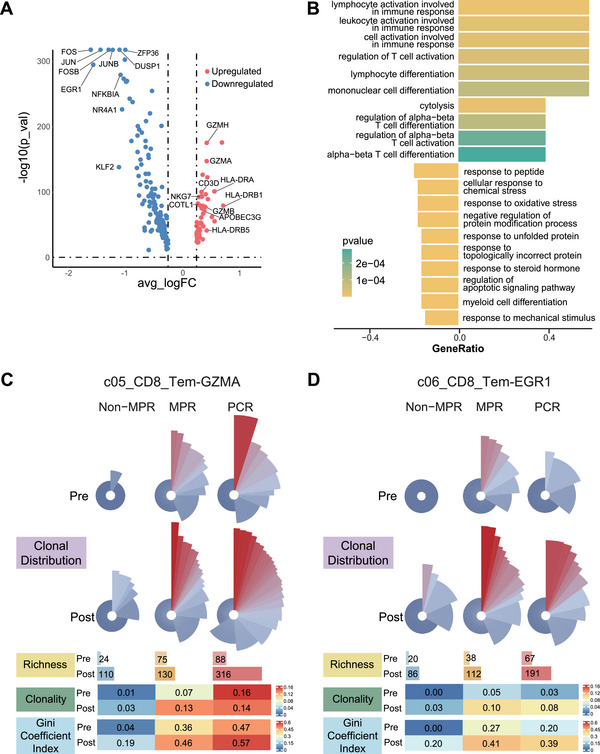
Distinct clonal profiles of two CD8+ effector memory T cell subsets. (A) Volcano map of differentially expressed genes between c05_CD8_Tem‐GZMA and c06_CD8_Tem‐EGR1. Benjamini–Hochberg adjusted Wilcoxon rank‐sum test, two‐sided. (B) Gene ontology analysis of the enrichment of specific pathways in c05_CD8_Tem‐GZMA and c06_CD8_Tem‐EGR1. (C and D) TCR measurements of clonal distribution of c05_CD8_Tem‐GZMA (C) and c06_CD8_Tem‐EGR1 (D), stratified by neoadjuvant efficacy and treatment time point.

We functionally compared the two effector memory subsets (c05 and c06) and found that c05 was more enriched in cytotoxicity and tumor immune response activation, while c06 showed enrichment in AP‐1 associated T‐cell differentiation (Figure 3A,B). The rose plot further confirmed that the TCR repertoire in c05 was more expanded at both pre‐ and post‐treatment conditions compared to c06 (Figure 3C,D). Notably, at pre‐treatment, responsive patients already exhibited a higher proportion of highly expanded TCR clones in c05 (shown in red) compared to the nonresponsive patient (Figure 3C,D).

Taken together, these findings highlight the specific clonal expansion of CD8^+^ T cells in responsive tumors and CD4^+^ Treg cells in nonresponsive patients, suggesting their potential roles in affecting the efficacy of neoadjuvant immunochemotherapy.

### 2.3 Special Clonal Expansion Patterns of CD8^+^ Effector Memory T Cells in Responsive Tumors

To further investigate the TCR clonal patterns in responsive tumors, we categorized TCR clones based on changes in their copy number between pre‐ and post‐treatments samples: novel nonexpanded clones (pre = 0, post = 1), novel expanded clones (pre = 0, post ≥ 2), and persistent expanded clones (pre ≥ 1, post ≥ 2, post > pre).

**FIGURE 4:**
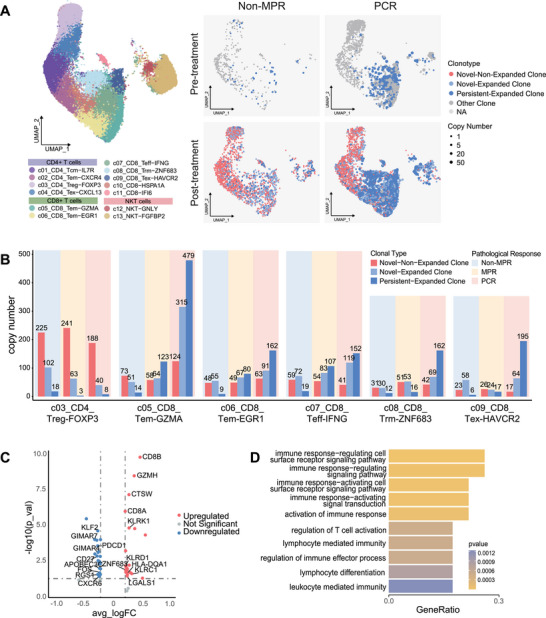
Special clonal expansion patterns of CD8+ effector memory T cells in responsive tumors. (A) UMAP visualization of selected T cells stratified by neoadjuvant efficacy and treatment time point. Each dot corresponds to one single cell, sized by clone size, colored by specific clonotypes indicating novel expanded clones (light blue), persistent expanded clones (dark blue), and novel nonexpanded clones (red). (B) Bar plot of post‐treatment copy number of specific clonotypes stratified by T‐cell cluster and neoadjuvant efficacy. The bar height represents the mean value. The bar color indicates diverse clonotypes. (C and D) Volcano map of differentially expressed genes between persistent expanded plus novel expanded clones and nonexpanded clones (C), and gene ontology analysis of the enrichment of specific pathways in c05_CD8_Tem‐GZMA post‐treatment PCR tumors (D).

As shown in UMAP, PCR and non‐MPR tumors exhibited distinct TCR clonal distributions (Figure 4A). Prior to treatment, PCR tumors already contained plenty of persistent expanded clones, which were rarely observed in non‐MPR tumors. After treatment, PCR tumors showed further enrichment in both persistent expanded and novel expanded clones, particularly within certain CD8^+^ T‐cell subsets. Quantitative analysis of TCR clonotypes within individual clusters revealed that the persistent expanded and novel expanded clones were preferentially enriched in the c05_CD8_Tem‐GZMA subset (Figure 4B). These clones exhibited high expression of cytotoxic genes (GZMH, KLRD1, KLRC1, and CTSW) and were associated with pathways involved in T‐cell activation, cytotoxicity, and anti‐tumor immunity (Figure 4C,D).

These findings suggest that a potential mechanism underlying the responsiveness to neoadjuvant immunochemotherapy may be mediated by a subset of highly expanded CD8^+^ effector memory T cells with cytotoxic and anti‐tumor functions. Not only do these cells respond positively to treatment, but some of these T‐cell clones were already existing and even expanded prior to therapy.

### 2.4 Enhanced Functional Profiles of Tregs in Nonresponsive Tumors

**FIGURE 5:**
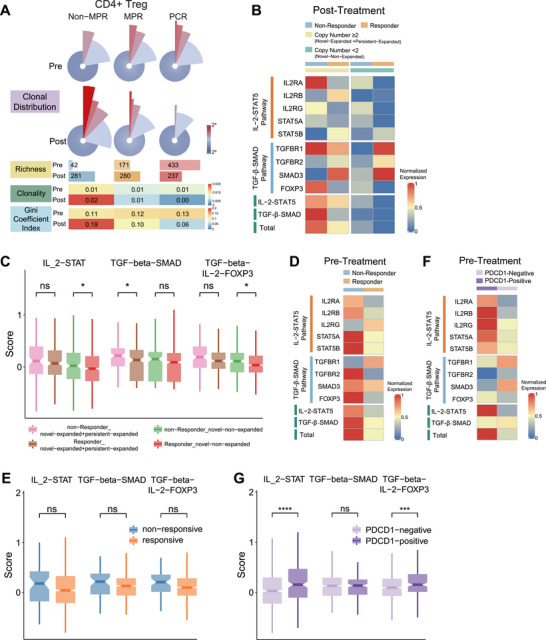
Enhanced functional profiles of CD4+ Tregs in nonresponsive tumors. (A) TCR measurements of clonal distribution of Treg cells stratified by neoadjuvant efficacy and treatment time point. (B) Heatmap of *z*‐score normalized mean expression of IL‐2‐STAT5 and TGF‐β‐SMAD pathway‐associated genes in Treg cells stratified by neoadjuvant efficacy and clone size for post‐treatment tumors. IL‐2‐STAT5 and TGF‐β‐SMAD pathway scores are calculated as *z*‐score normalized mean AUC score of single cells in each subgroup. (C) Box plot of mean AUC score of IL‐2‐STAT5 and TGF‐β‐SMAD pathway in post‐treatment Tregs. Center line with notch indicates the median value, lower and upper hinges represent the 25th and 75th percentiles, respectively, and whiskers denote 1.5× interquartile range. *p < 0.05; **p < 0.01; ***p < 0.001; ****p < 0.0001; ns, not significant; two‐sided unpaired *t*‐test. (D) Heatmap of *z*‐score normalized mean expression of IL‐2‐STAT5 and TGF‐β‐SMAD pathway‐associated genes in Treg cells stratified by neoadjuvant efficacy for pre‐treatment tumors. (E) Box plot of mean AUC score of IL‐2‐STAT5 and TGF‐β‐SMAD pathway in pre‐treatment Tregs stratified by neoadjuvant efficacy for pre‐treatment tumors. (F) Heatmap of *z*‐score normalized mean expression of IL‐2‐STAT5 and TGF‐β‐SMAD pathway‐associated genes in Treg cells stratified by PDCD1 expression for pre‐treatment tumors. (G) Box plot of mean AUC score of IL‐2‐STAT5 and TGF‐β‐SMAD pathway in pre‐treatment Tregs stratified by PDCD1 expression for pre‐treatment tumors.

As Treg cells exhibited clonal expansion after neoadjuvant therapy, we next investigated whether these clonal alterations were related to therapeutic efficacy. As shown in Figure 5A, TCR clonotype richness of Treg cells at post‐treatment was inversely associated with pathological response to neoadjuvant therapy. Consistently, quantitative analyses using clonality and the Gini coefficient index further supported these observations (Figure 5A). Compared with responsive tumors, nonresponsive tumors exhibited a higher abundance of both novel expanded clones (light blue bar in Figure 4B) and persistent expanded clones (dark blue bar in Figure 4B), whereas the amount of novel nonexpanded clones was comparable between the two groups.

We next examined the expression of genes related to IL‐2‐STAT5 and TGF‐β/‐SMAD signaling pathways in these expanded Treg clones, as both pathways have been reported to play critical roles in promoting Treg development [17]. Compared with responsive tumors, nonresponsive tumors exhibited higher expression of IL‐2‐STAT5 and TGF‐β/SMAD‐associated genes in both expanded Treg clones (copy number > 2) and novel nonexpanded Treg clones (copy number = 1) at post‐therapy (Figure 5B,C). Notably, a similar trend of enhanced pathway activation in nonresponsive tumors was already present prior to treatment, although the difference did not reach statistical significance (Figure 5D,E).

Given previous reports that PD‐1 expression levels can influence the efficacy of PD‐1 blockade [18], we further investigated whether PD‐1 expression on Tregs was associated with clonal dynamics during treatment and response to neoadjuvant therapy. CD4^+^ Tregs were stratified into PDCD1‐negative (PDCD1 expression = 0) and PDCD1‐positive (PDCD1 expression > 0) subsets, and differences in their cellular proportions and functional activation status were compared before and after treatment.

At the post‐treatment stage, we observed a trend toward a higher proportion of PDCD1‐positive Tregs among total CD4^+^ T cells in nonresponsive tumors (Figure S6C), a finding that was further validated by fluorescence flow cytometry in an independent cohort (Figure S6D,E). In contrast, no comparable difference was observed at the pre‐treatment stage (Figure S6F). However, when examining the functional activation status of Tregs at pre‐treatment, PDCD1‐positive Tregs exhibited significantly higher activity in both IL‑2‑STAT5 and TGF‑β/SMAD pathways (Figure 5F,G).

Taken together, these findings suggest that PD‐1 blockade stimulates the activation, novel clonal generation, and expansion of CD4^+^ Tregs through engagement of downstream IL‐2‐STAT5 and TGF‐β/SMAD signaling pathways, thereby ultimately compromising the therapeutic efficacy of neoadjuvant immunochemotherapy.

## 3 Discussion

In this study, we integrated paired tumor specimens with scRNA‐seq and scTCR‐seq to delineate how T‐cell reprogramming and TCR clonal dynamics influence responses to neoadjuvant immunochemotherapy. We found that responsive treatment drives persistent expansion of pre‐existing and newly emerging CD8^+^ Tem clones, while reducing clonal proliferation of Treg cells. These shifts collectively create a more activated intratumoral microenvironment, enhancing antitumor cytotoxicity.

Existing evidence from peripheral blood or unpaired tissues has suggested therapy‐associated cytotoxic T‐cell expansion, yet such approaches are limited by immune heterogeneity across compartments and lack of direct intratumoral evidence [11, 12, 13, 14]. By leveraging paired tumor samples, our study provides more robust evidence of microenvironment remodeling and highlights key immune determinants of responses to neoadjuvant immunochemotherapy. Tumors characterized by abundant baseline CD8^+^ Tem expansion and low Treg levels were more likely to achieve favorable outcomes. Moreover, therapy‐induced augmentation of CD8^+^ Tem clonal expansion, together with functional modulation of Tregs, plays a central role in shaping neoadjuvant efficacy.

Previous study of neoadjuvant anti‐PD‐1 monotherapy highlighted MANA‐specific CD8^+^ T cells with transcriptional features of Trm cells [10], a tissue‐resident subset that provides rapid local immune protection. Although adding chemotherapy did not markedly enhance CD8^+^ Trm reprogramming in our cohort, responsive tumors still showed enriched Trm infiltration and TCR clonal expansion after therapy. This may reflect the prolonged treatment cycles in immune monotherapy, which predominantly engages Trm cells, whereas immunochemotherapy with only two 3‐week treatment cycles more effectively activates CD8^+^ Tem cells. Consistently, we observed notable TCR clonal sharing between Trm and Tex subsets, and trajectory analyses suggested a Tem‐to‐Teff transition contributing to treatment response.

A prior study in advanced NSCLC identified precursor‐exhausted CD8^+^ T cells as major contributors to immunotherapy response [19]. In line with this, we observed substantial TCR sharing between exhausted CD8^+^ T cells and the CD8^+^ Tem‐GZMA subset, suggesting a developmental continuum. Differences in dominant T‐cell subsets between early‐stage and advanced disease may largely reflect treatment duration: after only two cycles of neoadjuvant immunochemotherapy, intratumoral T cells retain effector and resident memory functions, enabling rapid response, whereas prolonged therapy in advanced settings drives progressive exhaustion. Our trajectory analysis further supports a Tem‐to‐Tex transition underlying these observations.

Treg cells exert negative immunomodulatory effects, maintain immune tolerance, and preserve immune homeostasis. Although prior studies have suggested that Tregs influence therapeutic efficacy, their unpaired design leaves key questions unsolved: Are the increased Tregs observed in surgical specimens pre‐existing, or are they induced by neoadjuvant immunotherapy? And why, or through what mechanisms, might neoadjuvant immunochemotherapy specifically activate Tregs? Using paired TCR‐based analyses, we provide critical evidence that CD4^+^ Tregs arise from both in situ clonal expansion and newly generated clones, supported by enhanced IL‐2 and SMAD pathway activity. However, the proportion of PDCD1^+^ Tregs before treatment did not show differences between responders and nonresponders and exhibited substantial intra‐group variability (Figure S6F). This may be attributable to the inherent limitations of pre‐treatment biopsy samples, including limited cell numbers and sampling bias. From another perspective, these findings also suggest that biomarkers predictive of therapeutic response derived from biopsy‐based sampling may be unstable and subject to intrinsic limitations.

Our study has several limitations. First, the small sample size restricts the statistical power of our analyses, although the paired‐sample design offers more direct and convincing evidence of dynamic immune changes. In addition, pre‐treatment tissues were obtained through small biopsies, yielding limited materials and preventing comprehensive baseline flow cytometry and whole‐exome sequencing. Second, our findings rely heavily on single‐cell analyses with limited functional validation, and the lack of suitable animal models further constrains mechanistic investigation. Future studies with larger clinical cohorts and multi‐level validation—spanning cellular, animal, genomic, and proteomic analyses—will be essential to substantiate our findings.

In conclusion, by integrating paired pre‐ and post‐treatment specimens, our study delineates the tumor microenvironment landscape and TCR clonal profiling, revealing T‐cell immune reprogramming and TCR dynamics that underlies responses to neoadjuvant immunochemotherapy. These findings provide a deeper understanding of the mechanisms driving therapeutic efficacy.

## 4 Methods

### 4.1 Patients and Sample Collection

Patients with previously untreated, pathologically confirmed stage IIB‐IIIB NSCLC were enrolled in this prospective, single‐arm study conducted at the Second Affiliated Hospital of Zhejiang University School of Medicine between March 2021 and September 2021. Baseline staging was determined according to the eighth edition of the AJCC Cancer Staging Manual [20]. Primary tumor samples were obtained by bronchoscopye or percutaneous fine‐needle aspiration biopsy prior to therapy (pre‐treatment). All patients received two 3‐week cycles of neoadjuvant immunochemotherapy consisting of camrelizumab (PD‐1 inhibitor, 200 mg; Jiangsu HengRui Medicine Co., Ltd., China), carboplatin (area under the curve [AUC] 5 mg∙mL^−1^∙min^−1^), and pemetrexed (500 mg∙m^−2^) for nonsquamous NSCLC patients with lung nonsquamous cell carcinoma/nab‐paclitaxel (100 mg∙m^−2^) for those with lung squamous cell carcinoma. Post‐treatment tumor samples were collected at the time of surgical resection. Pathological responses were assessed on hematoxylin‐ and eosin‐stained slides by estimating the percentage of residual viable tumor relative to necrosis, fibrosis, and inflammation across the tumor cross‐section [21, 22]. Pathologic complete response (PCR) was defined as the absence of viable tumor cells in both the primary tumor and sampled regional lymph nodes. Major pathologic response (MPR) was defined as ≤ 10% viable tumor cells in the primary tumor, regardless of lymph node status. Cases with > 10% viable tumor cells in the primary tumor were classified as non‐MPR.

The study protocol was approved by the Institutional Review Boards and ethics committees (No. 2021‐0378) and was registered on ClinicalTrials.gov (NCT ChiCTR2100045803). Written informed consent was obtained from all participants.

### 4.2 Sample Tissue Processing

Immediately after collection, all samples were preserved in RNAlater and stored at 4°C to minimize RNA degradation. The interval from biopsy to processing was kept within 1 h for all samples to ensure consistency. Fresh tumor tissues were cut into 1–3 mm^3^ fragments in RPMI‐1640 medium (Gibco) supplemented with 10% fetal bovine serum (FBS, Gibco) and transferred into gentleMACS C tubes (Miltenyi Biotec). Tissues were enzymatically digested using the Tumor Dissociation Kit (Miltenyi Biotec) on a gentleMACS Dissociator (Miltenyi Biotec) for 60 min at 37°C.

The resulting cell suspensions were filtered through a 70 µm cell strainer (BD Falcon) and centrifuged at 300 × *g* for 10 min. After removing the supernatant, pellets were incubated in 1× red blood cell lysis buffer (Miltenyi Biotec) for 2 min at room temperature. Cells were then centrifuged at 300 × *g* for 10 min, and the supernatant was discarded. After two washes with PBS, the cell pellets were resuspended in PBS containing 1% FBS. The final concentration of single‐cell suspensions was adjusted to 700–1200 cells/µL.

### 4.3 Single‐Cell RNA and TCR Sequencing

Single‐cell suspensions were combined with gel beads carrying 10× barcodes and an oil surfactant to generate gel beads‐in‐emulsions (GEMs) using the microfluidic double‐cross system. Within each GEM, barcodes were released upon gel bead dissolution, while mRNAs were liberated through cell lysis and subsequently reverse transcribed to produce barcoded cDNAs. After breaking the oil emulsion, cDNAs from all GEMs were pooled and PCR‐amplified for library construction.

scRNA‐seq libraries were generated using the Chromium Single Cell 5′ Library & Gel Bead Kit (10× Genomics). TCR sequencing libraries were prepared using the Chromium Single Cell V(D)J Enrichment Kit, Human T Cell (10x Genomics), together with the Chromium Single Cell 5′ Library Construction Kit (10× Genomics), following the manufacturer's standard protocols. Sequencing was performed on an Illumina HiSeq X Ten platform with 150‐bp paired‐end reads.

### 4.4 Single‐Cell RNA Sequencing Data Processing and Analysis

#### 4.4.1 Quality Control of Single‐Cell RNA Sequencing

scRNA‐seq data were aligned and quantified using Cell Ranger v3.1 against the GRCh38 human reference genome. Additional downstream quality control steps were performed to remove low‐quality cells based on the following criteria: (1) cells with fewer than 250 or more than 6500 detected genes, indicative of empty droplets or multiplets; and (2) cells with > 25% mitochondrial gene counts. Genes detected in fewer than three cells were also excluded. Doublets removal was performed using the *Scrublet* algorithm [23], which simulates artificial doublets and eliminates high‐confidence true doublets based on gene expression similarities, thereby ensuring the robustness of subsequent analyses.

#### 4.4.2 Clustering and Annotation

Raw counts were normalized using the *NormalizeData* function in *Seurat* []. Highly variable genes (HVGs; *n* = 3000) were identified with *FindVariableFeatures*. Data were scaled using *ScaleData*, and 50 principal components (PCs) were computed using *RunPCA*. Batch effects were corrected using *RunHarmony*. A shared nearest‐neighbor (SNN) graph was constructed using the top 20 PCs with *FindNeighbors*, and clustering was performed using *FindClusters* at a resolution of 1.0. Nonlinear dimensionality reduction for visualization was conducted using *RunUMAP*.

#### 4.4.3 Differential Expression Analysis

Differentially expressed genes (DEGs) for each cluster were identified using *Seurat*’s *FindMarkers* and *FindAllMarkers* functions. Significant DEGs were defined as those with an absolute log_2_ fold change > 0.25 and an adjusted *p* value < 0.05 based on the Benjamini–Hochberg correction.

#### 4.4.4 Copy Number Variation (CNV) Analysis

To distinguish malignant epithelial cells, CNV analysis was performed using *inferCNV* [15], with T cells serving as an internal nonmalignant reference. Genome‐wide expression profiles of epithelial cells (the observation group) were compared against this reference using the default hidden Markov model, alongside denoising and an expression cutoff value of 0.1 to infer CNV events. Clustering of CNV profiles enabled the identification of epithelial subpopulations displaying widespread, high‐amplitude CNV alterations, which were classified as malignant.

#### 4.4.5 T‐Cell Subgroup Analysis

To quantify the distribution of T‐cell subsets, we calculated the proportion of each subset in all samples collected pre‐ and post‐treatment. Specimens containing fewer than 20 T cells were excluded to avoid biases arising from imprecise estimates.

#### 4.4.6 Trajectory Inferences With Monocle 3, Slingshot, and RNA Velocity Analysis

Single‐cell trajectory analysis was performed using *Monocle 3* [25] to reconstruct differentiation pathways and infer pseudotemporal ordering. Preprocessed data were embedded using *UMAP* [26] and clustered with the *Leiden* algorithm. A trajectory graph was then learned to model cell‐state transitions. Root cells were designated based on the expression of early‐stage markers (e.g. *TOP2A* and *MKI67*), enabling pseudotime ordering using the *order_cells* function. Genes whose expression correlated with pseudotime were identified for downstream analyses.

Trajectory inference was further conducted using the *Slingshot* algorithm [27] implemented in R. The UMAP embedding and cluster labels obtained from unsupervised clustering were provided as input. Slingshot inferred the global lineage structure by constructing a minimum spanning tree (MST) across cluster centroids, from which the starting cluster was determined. Principal curves were fitted simultaneously to model gene‐expression changes along pseudotime. The resulting pseudotime values for each cell were used for downstream analyses of gene‐expression dynamics across differentiation trajectories.

RNA velocity analysis was implemented to characterize dynamic transcriptional changes. Spliced and unspliced Unique Molecular Identifiers (UMIs) were quantified using the *velocity* Python package [28]. Count matrices were normalized, and HVGs were identified. Moments of normalized spliced and unspliced counts were then calculated for each cell, and a velocity graph was constructed using cosine similarity. RNA velocity vectors were projected onto the UMAP embedding to visualize the inferred cellular dynamics.

#### 4.4.7 Cell–Cell Interaction Analysis With CellPhoneDB

Cell–cell communication was analyzed using *CellPhoneDB* analysis [29]. Gene expression matrices were used as input to compute the mean expression of ligand–receptor pairs within each cluster. Statistical significance was assessed using a permutation‐based framework involving thousands of random shuffles of cluster labels. Ligand‐receptor interactions with *p* < 0.05 and biological relevance were retained for downstream interpretation.

### 4.5 Single‐Cell TCR Data Processing and Analysis

#### 4.5.1 Quality Control of Single‐Cell TCR Sequencing

Raw TCR sequencing data were aligned and assembled using Cell Ranger v3.1 (10x Genomics) with the GRCh38 human V(D)J reference genome. Quality control and clonotyping were performed in two sequential steps. First, at the cell level, we retained only cells that passed scRNA‐seq quality control and contained productive, full‐length TCR α and β chains. Second, at the clonotype level, the dominant productive α/β‐chain pair detected in each cell was defined as its functional TCR receptor. Each unique α/β‐chain combination was designated as a clonotype, and cells sharing the same α/β pair were assigned to the same T‐cell clone. Only cells harboring a productive α/β‐chain pair were included in downstream analyses.

#### 4.5.2 Diversity and Clonality Measures of T‐Cell Repertoire

T‐cell repertoire diversity was quantified using richness, defined as the number of unique TCR sequences. Evenness—reflecting the distribution of clonotype frequencies—was assessed using clonality and the Gini coefficient. Clonality was calculated as 1 minus Pielou's evenness, where Pielou's evenness is the Shannon entropy normalized by the natural logarithm of TCR richness:

$$\mathrm{Clonality}\;=1-\frac{-\sum_{i=1}^{N}p_{i}\log_{2}\left(p_{i}\right)}{\log_{2}\left(p_{i}\right)},$$

where $p_{i}$ is the proportional abundance of the ith clonotype and *N* is the total number of clonotypes.

The Gini coefficient, which is independent of richness, was calculated as:

$$\mathrm{Gini}\;\mathrm{coefficient}\;=\frac{\sum_{i=1}^{N}\sum_{j=1}^{N}\left|p_{i}-p_{j}\right|}{2N^{2}\bar{p}}\;,$$

where $p_{i}$ and $p_{j}$ are the frequencies of the ith and jth clonotypes, respectively, $\bar{p}$ denotes the mean clone frequency, and *N* is the total number of clonotypes.

#### 4.5.3 Classification of Expanded and Novel Nonexpanded Clonotypes

Based on the presence and abundance of TCR clonotypes before and after treatment, clonotypes were categorized into the following groups:

1) Expanded clones: They include clonotypes with higher abundance post‐treatment than pre‐treatment (post ≥ 2, post > pre), which included both novel expanded clones (undetectable before treatment but expanded after treatment; pre = 0, post ≥ 2) and persistent expanded clones (present before treatment and further expanded after treatment; pre ≥ 1, post ≥ 2).

2) Novel nonexpanded clones: They include clonotypes absent before treatment but detected at low abundance after treatment (pre = 0, post = 1).

Clonotypes that did not meet the criteria for the above categories were classified as other clones.

### 4.6 Flow Cytometry Analysis of an Independent Validation Cohort

We enrolled an additional independent cohort of NSCLC patients who underwent neoadjuvant immunochemotherapy and collected surgically resected tumor tissue samples after treatment. The cohort consisted of four nonresponders (non‐MPR) and four responders (PCR). Tissue samples were processed into single‑cell suspensions through mechanical dissociation and enzymatic digestion, followed by staining with a pre‑optimized antibody panel for surface and intracellular markers. Data were acquired by flow cytometry to characterize GZMA^+^ CD8^+^ Tem cells and PD‑1^+^ Treg cells in the tumor microenvironment.

The gating strategy was performed as follows. First, target cells were preliminarily gated based on the FSC‑A/SSC‑A scatter plot, and doublets were excluded using FSC‑H/FSC‑A. Subsequently, lymphocytes were identified within the CD45‑positive population by gating on cells with the lowest side‑scatter signal (SSC‑A[low]). Next, CD3^+^ cells were selected to define total T cells, from which CD8^+^ T cells were further gated. Among CD8^+^ T cells, differentiation status was determined according to the expression patterns of CD45RA and CCR7. The CD45RA^−^CCR7^−^ subset was defined as effector memory T cells (Tem), and Granzyme A (GZMA) expression was analyzed within this subset to identify GZMA^+^ CD8^+^ Tem cells. For the analysis of Tregs, Tregs were gated from the CD3^+^CD4^+^ T‑cell population based on high CD25 expression together with low CD127 expression. PD‑1 expression was then examined within the Treg gate to quantify the proportion of PD‑1^+^ Treg cells.

### 4.7 Statistical Analysis

Statistical analyses were performed using GraphPad Prism (version 9.0) for experimental data and a combination of R (version 3.6.1), RStudio (version 3.5.3), and Python (version 3.7.4) for sequencing data processing and downstream analyses. Comparisons between groups were conducted using *χ* ^2^ tests or Fisher's exact test for categorical variables, and Student's *t*‐tests or Wilcoxon rank‐sum (Mann‐Whitney *U*) tests for continuous variables, as appropriate. Paired measurements were analyzed using paired *t*‐tests. A *p*‐value <0.05 was considered statistically significant.

## Author Contributions

Yang Xia, Wei Chen, Weiwei Yin, and Wen Li contributed to conception and design of the study. Yang Xia and Wen Li provided resources and administrative support. Yang Xia, Rui Jin, Qiyuan Wang, Xiuzhen Li, Sai Zhang, and Guanxin Xu involved in sample collection and evaluation. Yang Xia, Rui Jin, Weina Lu, Kai Zhu, Peng Li, Jianan Li, Wei Chen, and Weiwei Yin contributed to data analysis and interpretation. Yang Xia and Rui Jin wrote the first draft of the manuscript with contribution from all authors. Rui Jin, Anhao Tian, and Yang Xia participated in the experimental procedures for flow cytometry validation cohort and contributed to the revision of data analysis. Yang Xia, Wei Chen, Weiwei Yin, and Wen Li critically reviewed or revised the manuscript for important intellectual content. All authors reviewed the drafts and the final version of the manuscript, and agree with its content and submission.

## Funding

This work was supported by the National Natural Science Foundation of China (82370028, 82422001 and 82102894), Development Project of Zhejiang Province's “Jianbing” and “Lingyan” (2026C02A1127), Noncommunicable Chronic Diseases‐National Science and Technology Major Project (2024ZD0528500), and the Fundamental Research Funds for the Central Universities (226‐2025‐00033).

## Ethics Statement

The clinical protocol was approved by the institutional review boards and ethics committees (No. 2021‐0378) and is registered on ClinicalTrials.gov (NCT ChiCTR2100045803). All participants provided written informed consent.

## Conflicts of Interest

Peng Li and Jianan Li are employees of Zhejiang Puluoting Health Technology Co., Ltd, but have no potential relevant financial or nonfinancial interests to disclose. The other authors have no conflicts of interest to declare.

## Supporting information




**Supporting file 1**: mco270690‐sup‐0001‐SupMat.pdf

## Data Availability

Data are available upon reasonable request. All data relevant to the study are included in the article or uploaded as Supporting Information, except for the raw data for single‐cell RNA and TCR sequencing that can be accessed in the National Genomics Data Center (https://ngdc.cncb.ac.cn/) under the accession number HRA004588 on request.
