# Supplementary material for: Dynamic T‐Cell Reprogramming Modulates the Treatment Outcome of Neoadjuvant Immunochemotherapy in Non‐Small‐Cell Lung Cancer
Source: MedComm (2020). 2026 Mar 18;7(4):e70690. doi: 10.1002/mco2.70690 (PMC13042455; doi:10.1002/mco2.70690)
Supplement: Supplementary file 1 — Supporting file 1: mco270690‐sup‐0001‐SupMat.pdf [file MCO2-7-e70690-s001.pdf]

Dynamic T Cell Reprogramming Modulates the Treatment Outcome of Neoadjuvant Immunochemotherapy in Non-Small-Cell Lung Cancer

Rui Jin, MS[1, #], Anhao Tian, PhD[2, #], Weina Lu, MS[1, 3, #], Qiyuan Wang, MS[4], Xiuzhen Li, MS[5], Sai Zhang, PhD, MD[6], Guanxin Xu, MD[6], Kai Zhu, BS[7], Peng Li, MS[8], Jianan Li, MS[8], Wei Chen, PhD[9, 10], Weiwei Yin, PhD[9, 11 *], Wen Li, MD[1,3, *], Yang Xia, PhD, MD[1, 3, *]

[1] Key Laboratory of Respiratory Disease of Zhejiang Province, Department of Respiratory and Critical Care Medicine, Second Affiliated Hospital of Zhejiang University School of Medicine, Hangzhou, Zhejiang, 310009, China.

[2] Department of Neurosurgery, Second Affiliated Hospital of Zhejiang University School of Medicine, Hangzhou, Zhejiang, 310009, China.

[3] Cancer Centre, Zhejiang University, Hangzhou, Zhejiang, 310058, China.

[4] Department of Radiology, Second Affiliated Hospital of Zhejiang University School of Medicine, Hangzhou, Zhejiang, 310009, China.

[5] Department of Pathology, Second Affiliated Hospital of Zhejiang University School of Medicine, Hangzhou, Zhejiang, 310009, China.

[6] Department of Thoracic Surgery, Second Affiliated Hospital of Zhejiang University School of Medicine, Hangzhou, Zhejiang, 310009, China.

[7] Wenzhou Medical University, Wenzhou, Zhejiang, 325035, China.

[8] Zhejiang Puluoting Health Technology Co., Ltd, Hangzhou, Zhejiang, 310009, China.

[9] Key Laboratory for Biomedical Engineering of Ministry of Education, College of Biomedical Engineering and Instrument Science, Zhejiang University, Hangzhou, Zhejiang, 310027, China.

[10] School of Basic Medical Science, Zhejiang University, Hangzhou, Zhejiang, 310058, China.

[11]Zhejiang Provincial Key Laboratory of Cardio-Cerebral Vascular Detection Technology and Medicinal Effectiveness Appraisal, College of Biomedical Engineering and Instrument of Science, Zhejiang University, Hangzhou, Zhejiang, China

[#] Those authors contributed equally.

[*] Corresponding Authors:

Yang Xia, MD, PhD

Department of Respiratory and Critical Care Medicine,

Second Affiliated Hospital, Zhejiang University School of Medicine,

Hangzhou, Zhejiang, 310009, China

Email: yxia@zju.edu.cn

Wen Li, MD

Department of Respiratory and Critical Care Medicine,

Second Affiliated Hospital, Zhejiang University School of Medicine,

Hangzhou, Zhejiang, 310009, China

Email: liwen@zju.edu.cn

Weiwei Yin, PhD

Key Laboratory for Biomedical Engineering of the Ministry of Education,

College of Biomedical Engineering and Instrument Science, Zhejiang University,

Hangzhou, Zhejiang, 310027, China.

Email: wwyin@zju.edu.cn

Table S1. Canonical Marker Genes of Major Cell Types

Characteristics	Canonical Marker Genes
**T/NK cells**	*CD3D, CD8A, CD4, NKG7*
**B cells**	*CD79A, MS4A1* (CD20)*, JCHAIN, MZB1*
**Myeloid cells**	*CD14, FCGR3A* (CD16)*, LYZ, S100A8, CPA3, KIT, TPSAB1*
**Fibroblasts**	*COL1A1, COL1A2, DCN, LUM*
**Epithelial cells**	*EPCAM, KRT14, KRT5, KRT7, KRT19*
**Endothelial cells**	*CLDN5, PECAM1* (CD31)*, VWF* (von Willebrand factor)*, CDH5*
**Proliferating cells**	*MKI67, STMN1, TOP2A*

Supplementary Figure Legend

Figure S1. Quality control information for single-cell sequencing data.

A. Number of read counts (upper), percentage of mitochondrial gene counts (middle), and number of genes (lower) in each sample. **B.** TCR counts in each sample. **C.** Elbow plot for principal component analysis.

Figure S2. Major cell subgroups in all cells.

A. UMAP visualization of all cells showing the formation of major subgroups, including T cells, B cells, Myeloid cells, fibroblasts, epithelial cells, malignant epithelial cells, endothelial cells, and proliferating cells. **B.** UMAP visualization of all cells stratified by neoadjuvant efficacy and treatment timepoint. **C.** Gene signature of major subgroups. **D.** Frequency of malignant cells (shown in red) detected by inferCNV. **E.** UMAP visualization of malignant cells (shown in red) detected by inferCNV.

Figure S3. Changes and distributions in major cell subgroups.

A-B. Frequency of major subgroups stratified by treatment timepoint (A) and neoadjuvant efficacy (B). **C.** Tissue preference of major subgroups stratified by neoadjuvant efficacy and treatment timepoint.

Figure S4. Gene and functional signatures of T cells.

A. Bubble heatmap of canonical feature genes of T cell subgroups. **B.** Feature gene expression of T cell subgroups. **C.** Functional status of T cell subgroups.

Figure S5. TCR clonal distribution and differentiation trajectory.

A. Histogram of TCR clonal distribution of T cell subgroups. **B.** RNA velocity analysis of T cell subgroups. **C.** Slingshot analysis of T cell subgroups.

Figure S6. Flow cytometry analysis of an additional independent validation cohort.

A. The gating strategy of flow cytometry for $GZMA^{+}$ $CD8^{+}$ T effector memory cells

from tumors. **B.** Boxplot of $GZMA^+$ $CD8^+$ T effector memory cell proportions among $CD8^+$ T cells in non-responders (non-MPR) and responders (PCR). **C.** The gating strategy of flow cytometry for $PD1^+$ Treg cells from tumors. **D.** Boxplot of $PD1^+$ Treg cell proportions among total T cells in non-responders (non-MPR) and responders (PCR). **E**. Boxplot of PD1-positive Treg cell proportions among $CD4^+$ T cells in non-responders and responders at post-treatment based on scRNA sequencing data. **F**. Boxplot of PD1-positive Treg cell proportions among $CD4^+$ T cells in non-responders and responders at pre-treatment based on scRNA sequencing data.

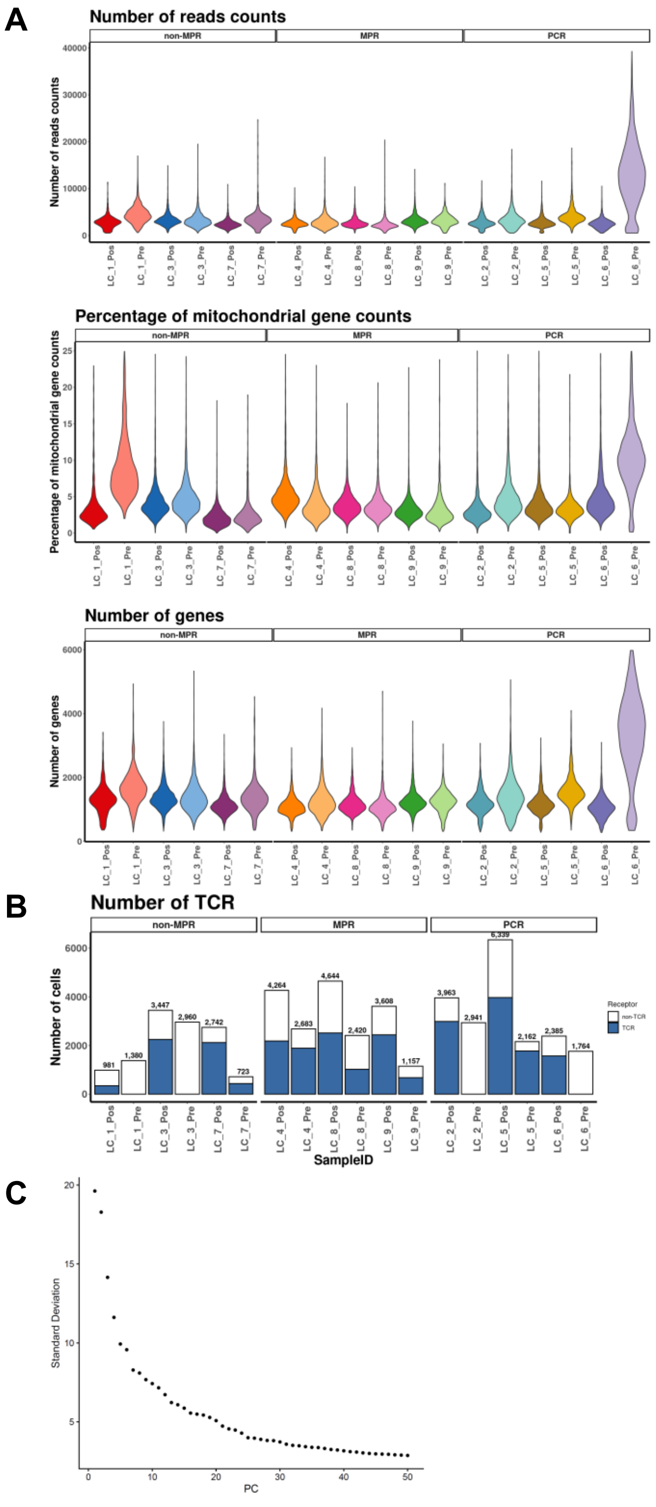

A
Number of reads counts
non-MPR
MPR
PCR
Number of reads counts
0
10000
20000
30000
40000
LC_1_Pos
LC_1_Pre
LC_3_Pos
LC_3_Pre
LC_7_Pos
LC_7_Pre
LC_4_Pos
LC_4_Pre
LC_8_Pos
LC_8_Pre
LC_9_Pos
LC_9_Pre
LC_2_Pos
LC_2_Pre
LC_5_Pos
LC_5_Pre
LC_6_Pos
LC_6_Pre
Percentage of mitochondrial gene counts
non-MPR
MPR
PCR
Percentage of mitochondrial gene counts
0
5
10
15
20
25
Number of genes
non-MPR
MPR
PCR
Number of genes
0
2000
4000
6000
B
Number of TCR
non-MPR
MPR
PCR
Number of cells
0
2000
4000
6000
981
1,380
3,447
2,960
2,742
723
4,264
2,683
4,644
2,420
3,608
1,157
3,963
2,941
6,339
2,162
2,385
1,764
Receptor
non-TCR
TCR
SampleID
C
Standard Deviation
5
10
15
20
0
10
20
30
40
50
PC

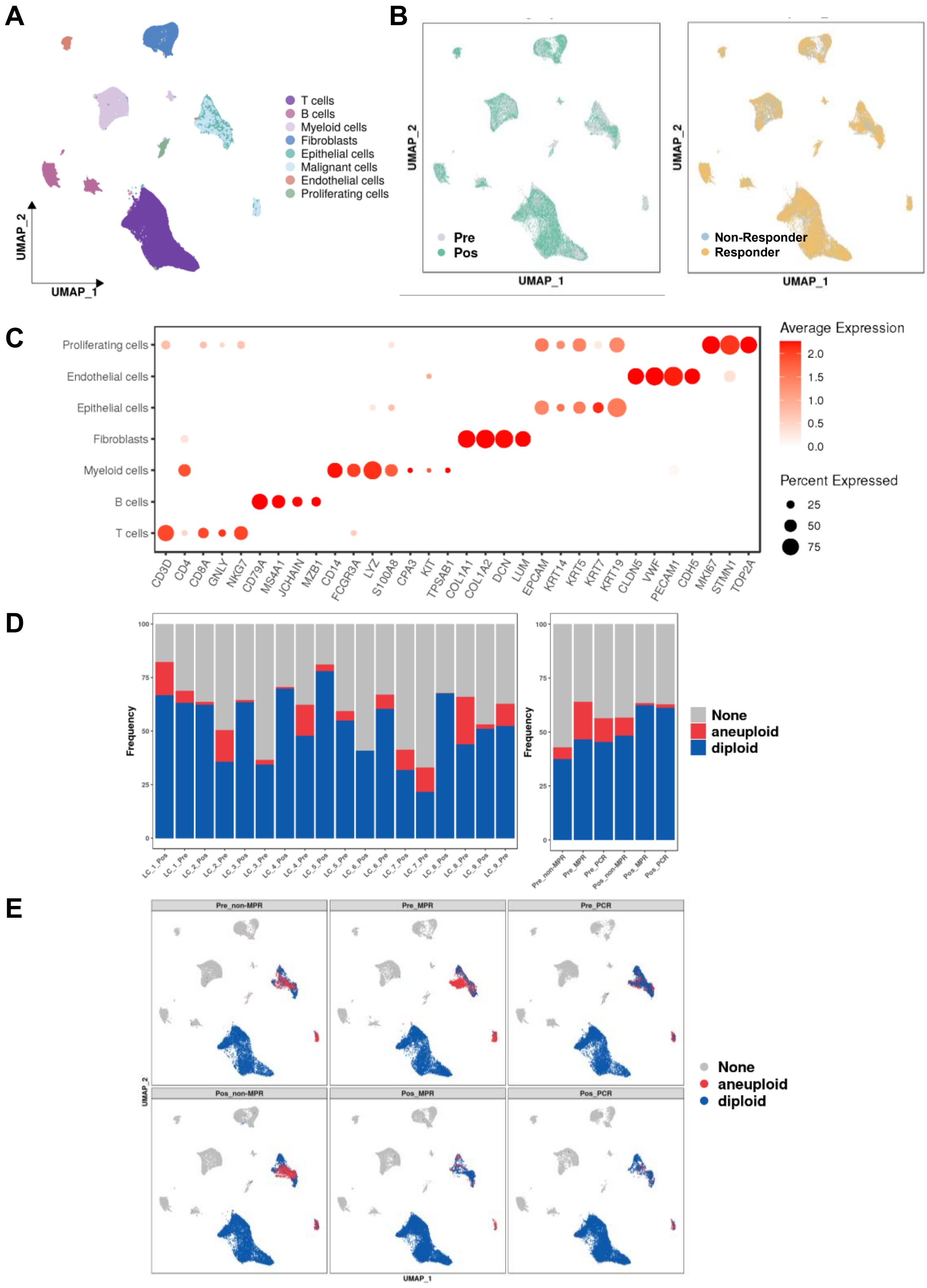
A
T cells
B cells
Myeloid cells
Fibroblasts
Epithelial cells
Malignant cells
Endothelial cells
Proliferating cells
UMAP_2
UMAP_1
B
Pre
Pos
Non-Responder
Responder
UMAP_2
UMAP_1
C
Proliferating cells
Endothelial cells
Epithelial cells
Fibroblasts
Myeloid cells
B cells
T cells
CD3D CD4 CD8A GNLY NKG7 CD79A MS4A1 JCHAIN MZB1 CD14 FCGR3A LYZ S100A8 CPA3 KIT TPSAB1 COL1A1 COL1A2 DCN LUM EPCAM KRT14 KRT5 KRT7 KRT19 CLDN5 VWF PECAM1 CDH5 MKI67 STMN1 TOP2A
Average Expression
2.0
1.5
1.0
0.5
0.0
Percent Expressed
25
50
75
D
Frequency
LC_1_Pos LC_1_Pre LC_2_Pos LC_2_Pre LC_3_Pos LC_3_Pre LC_4_Pos LC_4_Pre LC_5_Pos LC_5_Pre LC_6_Pos LC_6_Pre LC_7_Pos LC_7_Pre LC_8_Pos LC_8_Pre LC_9_Pos LC_9_Pre
Pre_non-MPR Pre_MPR Pre_PCR Pos_non-MPR Pos_MPR Pos_PCR
None
aneuploid
diploid
E
Pre_non-MPR
Pre_MPR
Pre_PCR
Pos_non-MPR
Pos_MPR
Pos_PCR
UMAP_2
UMAP_1
None
aneuploid
diploid

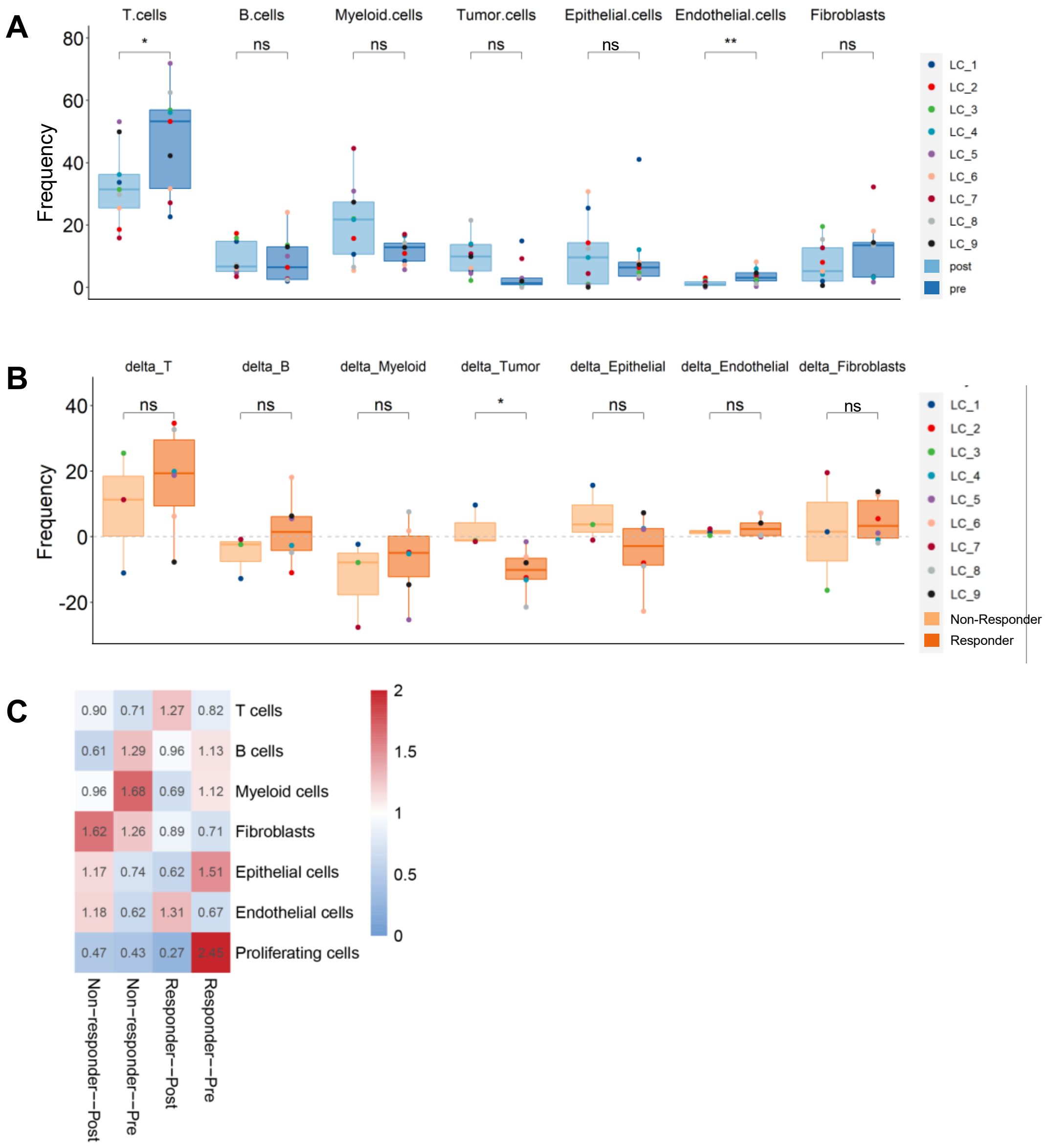

A
T.cells
B.cells
Myeloid.cells
Tumor.cells
Epithelial.cells
Endothelial.cells
Fibroblasts
Frequency
LC_1
LC_2
LC_3
LC_4
LC_5
LC_6
LC_7
LC_8
LC_9
post
pre
B
delta_T
delta_B
delta_Myeloid
delta_Tumor
delta_Epithelial
delta_Endothelial
delta_Fibroblasts
Non-Responder
Responder
C
0.90 0.71 1.27 0.82 T cells
0.61 1.29 0.96 1.13 B cells
0.96 1.68 0.69 1.12 Myeloid cells
1.62 1.26 0.89 0.71 Fibroblasts
1.17 0.74 0.62 1.51 Epithelial cells
1.18 0.62 1.31 0.67 Endothelial cells
0.47 0.43 0.27 2.45 Proliferating cells
Non−responder−−Post
Non−responder−−Pre
Responder−−Post
Responder−−Pre

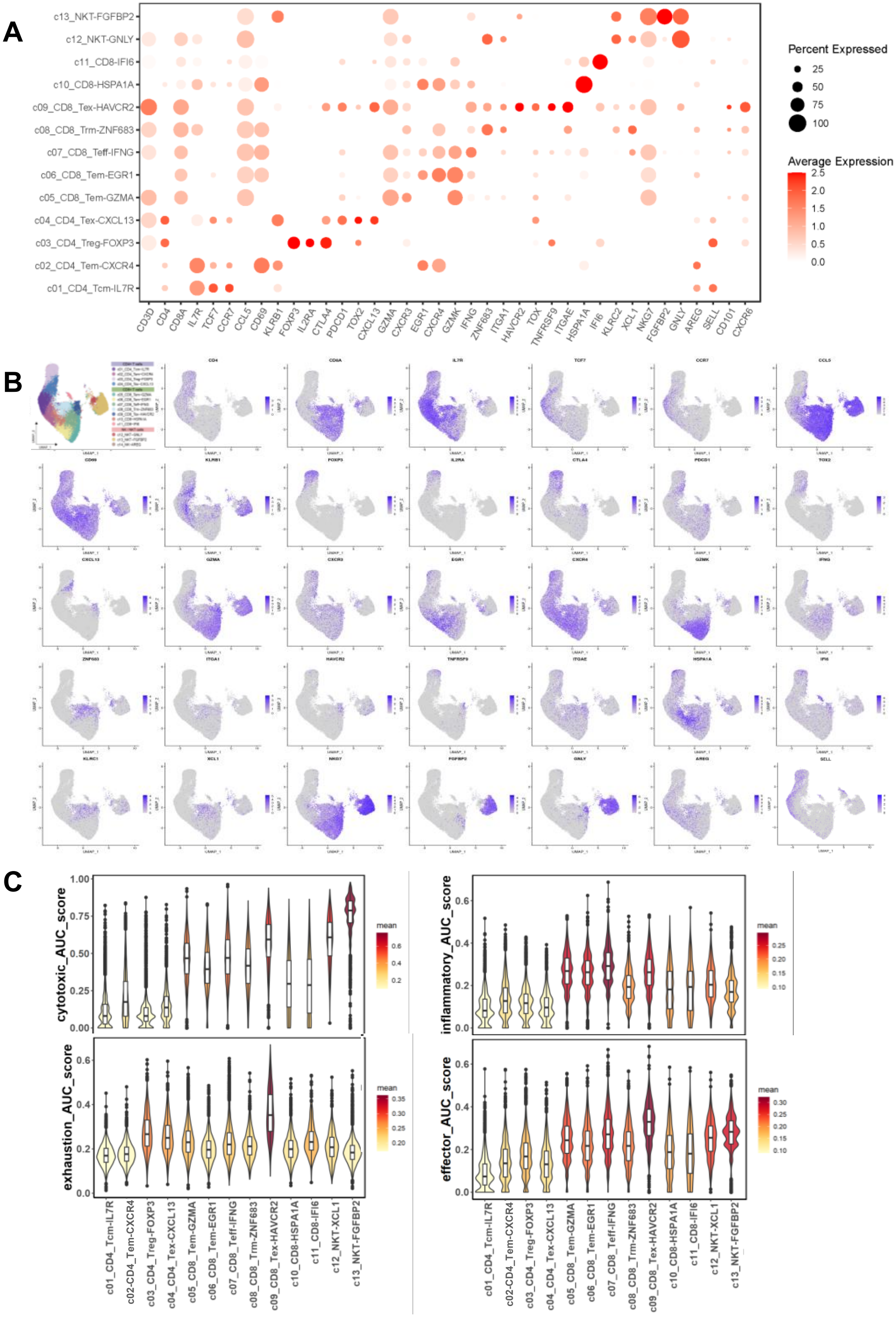

A
c13_NKT-FGFBP2
c12_NKT-GNLY
c11_CD8-IFI6
c10_CD8-HSPA1A
c09_CD8_Tex-HAVCR2
c08_CD8_Trm-ZNF683
c07_CD8_Teff-IFNG
c06_CD8_Tem-EGR1
c05_CD8_Tem-GZMA
c04_CD4_Tex-CXCL13
c03_CD4_Treg-FOXP3
c02_CD4_Tem-CXCR4
c01_CD4_Tcm-IL7R
CD3D CD4 CD8A IL7R TCF7 CCR7 CCL5 CD69 KLRB1 FOXP3 IL2RA CTLA4 PDCD1 TOX2 CXCL13 GZMA CXCR3 EGR1 CXCR4 GZMK IFNG ZNF683 ITGA1 HAVCR2 TOX TNFRSF9 ITGAE HSPA1A IFI6 KLRC2 XCL1 NKG7 FGFBP2 GNLY AREG SELL CD101 CXCR6
Percent Expressed
25
50
75
100
Average Expression
2.5
2.0
1.5
1.0
0.5
0.0
B
C
cytotoxic_AUC_score
inflammatory_AUC_score
exhaustion_AUC_score
effector_AUC_score
mean
c01_CD4_Tcm-IL7R
c02-CD4_Tem-CXCR4
c03_CD4_Treg-FOXP3
c04_CD4_Tex-CXCL13
c05_CD8_Tem-GZMA
c06_CD8_Tem-EGR1
c07_CD8_Teff-IFNG
c08_CD8_Trm-ZNF683
c09_CD8_Tex-HAVCR2
c10_CD8-HSPA1A
c11_CD8-IFI6
c12_NKT-XCL1
c13_NKT-FGFBP2

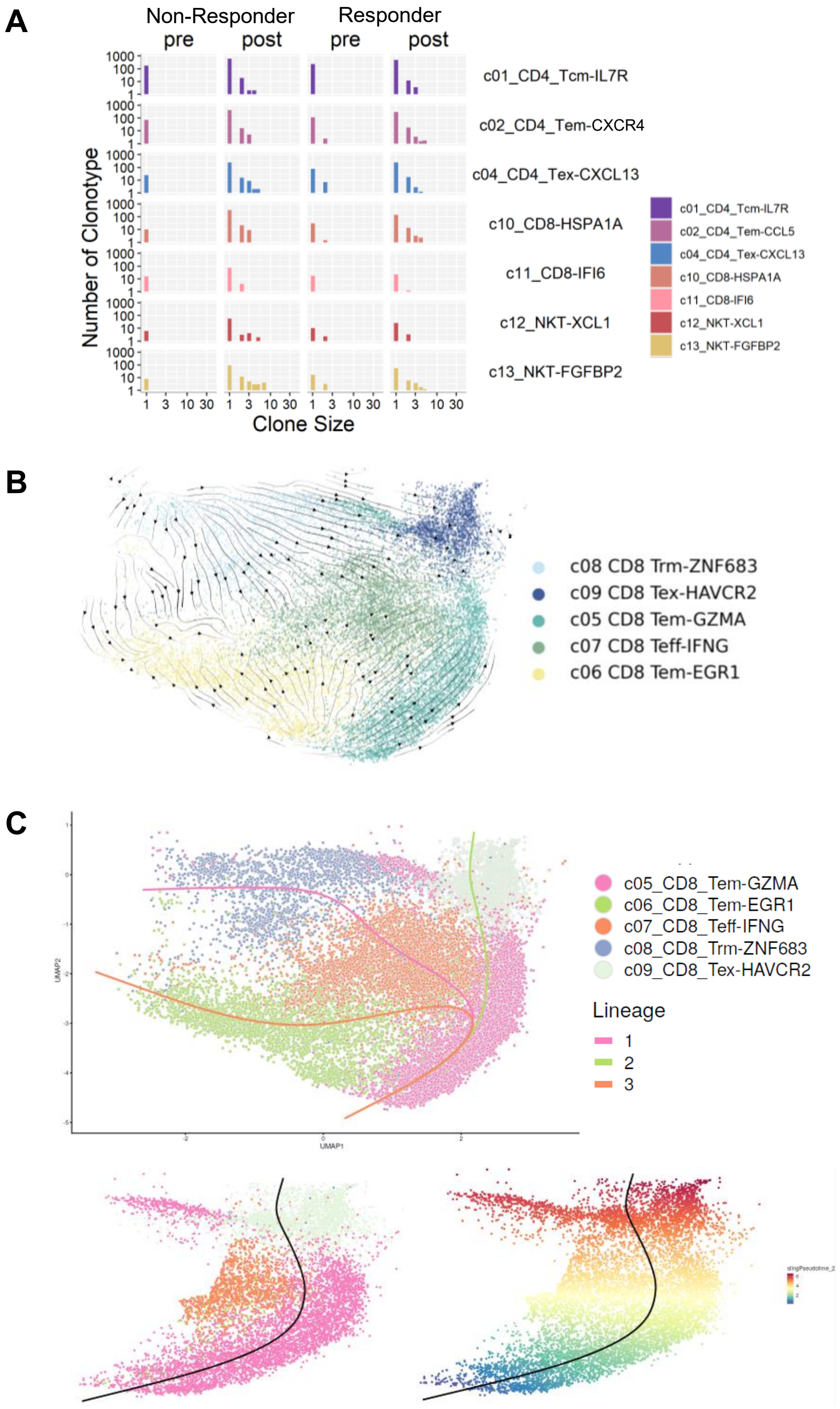
A
Non-Responder
Responder
pre
post
pre
post
Number of Clonotype
1000
100
10
1
1
3
10
30
Clone Size
c01_CD4_Tcm-IL7R
c02_CD4_Tem-CXCR4
c04_CD4_Tex-CXCL13
c10_CD8-HSPA1A
c11_CD8-IFI6
c12_NKT-XCL1
c13_NKT-FGFBP2
c01_CD4_Tcm-IL7R
c02_CD4_Tem-CCL5
c04_CD4_Tex-CXCL13
c10_CD8-HSPA1A
c11_CD8-IFI6
c12_NKT-XCL1
c13_NKT-FGFBP2
B
c08 CD8 Trm-ZNF683
c09 CD8 Tex-HAVCR2
c05 CD8 Tem-GZMA
c07 CD8 Teff-IFNG
c06 CD8 Tem-EGR1
C
c05_CD8_Tem-GZMA
c06_CD8_Tem-EGR1
c07_CD8_Teff-IFNG
c08_CD8_Trm-ZNF683
c09_CD8_Tex-HAVCR2
Lineage
1
2
3
UMAP1
UMAP2

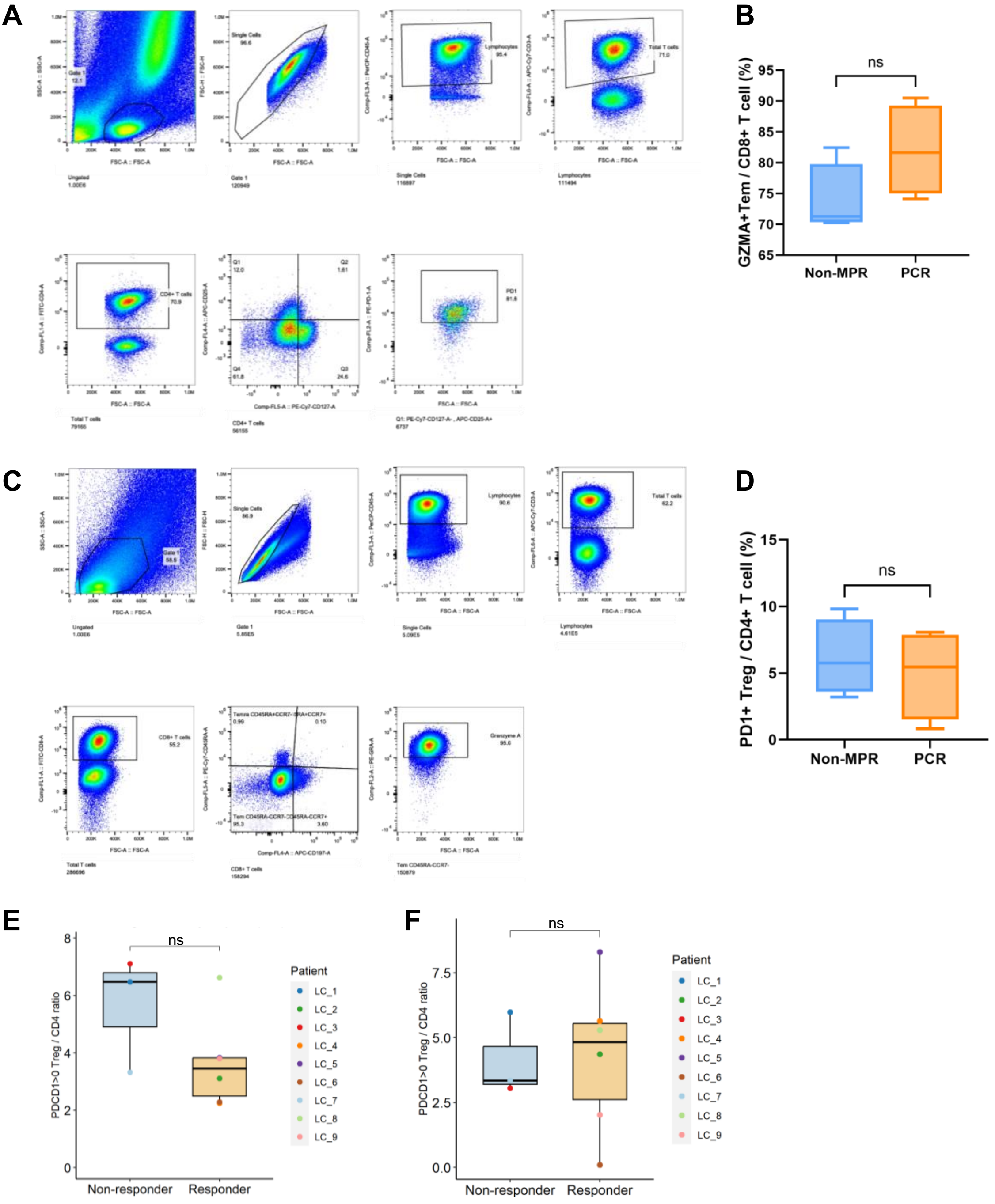

A
B
C
D
E
F
GZMA+Tem / CD8+ T cell (%)
95
90
85
80
75
70
65
ns
Non-MPR
PCR
PD1+ Treg / CD4+ T cell (%)
15
10
5
0
ns
Non-MPR
PCR
PDCD1>0 Treg / CD4 ratio
8
6
4
2
0
ns
Non-responder
Responder
Patient
LC_1
LC_2
LC_3
LC_4
LC_5
LC_6
LC_7
LC_8
LC_9
PDCD1>0 Treg / CD4 ratio
7.5
5.0
2.5
0.0
ns
Non-responder
Responder
Patient
LC_1
LC_2
LC_3
LC_4
LC_5
LC_6
LC_7
LC_8
LC_9
